# Dynamics of self-control during choice and post-choice consumption quantity

**DOI:** 10.3389/fpsyg.2024.1238780

**Published:** 2024-06-03

**Authors:** Ga-Eun Oh, Anirban Mukhopadhyay

**Affiliations:** ^1^Department of Marketing and International Business, Faculty of Business, Lingnan University, Tuen Mun, Hong Kong SAR, China; ^2^Faculty of Management, Bayes Business School, City, University of London, London, United Kingdom; ^3^Department of Marketing, School of Business and Management, Hong Kong University of Science and Technology, Hong Kong, Hong Kong SAR, China

**Keywords:** self-control, goal accessibility, food decision making, dietary restraint, food intake, exploratory research

## Abstract

Observed choices between options representing a relative vice and a relative virtue have commonly been used as a measure of eating self-control in the literature. However, even though self-control operations may manifest across the post-choice consumption stage, either similarly or in different ways from the choice stage, most prior research has ignored consumption quantity of the chosen option. While the behavior of choosing a virtue instead of a vice does manifest self-control, we examine how this plays out in post-choice consumption. Specifically, we find that when processing resources are limited, after having chosen a virtue food, unrestrained eaters ironically consumed greater quantities and therefore more calories than restrained eaters (Study 1). This reflects more persistent self-control in the post-choice consumption stage among restrained eaters than unrestrained eaters, and occurs because choosing a virtue lowers accessibility of the self-control goal among unrestrained eaters relative to restrained eaters (Study 2), thereby increasing intake of the virtuous food. In contrast, subsequent to having chosen a vice, unrestrained eaters and restrained eaters did not show any such difference in intake (Study 1) or goal accessibility (Study 2). Together, these results reveal that persistence of self-control in the post-choice consumption stage depends on individuals’ dietary restraint and their initial exercise of self-control in the choice decision. The mere act of choosing a virtue satisfies unrestrained eaters’ self-control goal and leads to increased food intake, whereas the same act keeps the same goal activated among restrained eaters who reduce intake of the chosen virtue. Put differently, persistent self-control across choice and quantity decisions is observed only when those with a dietary goal show successful self-control enactment in the choice stage. We therefore highlight that the operation of self-control can be dynamic within a consumption episode, and thus, choice and post-choice quantity are both informative of self-control.

## 1 Introduction

A consumer psychologist observes someone make a choice between a slice of chocolate cake and a bowl of fruit salad, a classic measure of self-control (Shiv and Fedorikhin, 1999). What does the choice reveal about the person’s self-control? Since chocolate cake [i.e., a “relative vice” (Wertenbroch, 1998)] is perceived as being relatively tastier but less healthy relative to fruit salad (“a relative virtue”), the choice of chocolate cake is usually interpreted as indicative of a lack of self-control. Correspondingly, a choice of fruit salad is attributed to the successful operation of self-control. Such inferences based on observing similar choices are a cornerstone of the literature on self-control–-a recent review of the relevant literature from 1998 to 2018 identified that over 120 published articles relied on such vice versus virtue choices to operationalize self-control (Vosgerau et al., 2020).^1^

Indeed, the choice of the virtue is indicative of self-control enactment as long as the choice task elicits conflict between desire and willpower – a critical and necessary component of self-control operations (Hoch and Loewenstein, 1991; Berman and Small, 2018). However, once consumers start eating the chosen virtue, will the self-control expressed in their virtue choice be sustained? For example, will it lead to lower consumption of the chosen virtue? What if a person chooses fruit salad while successfully giving up chocolate cake and therefore shows successful enactment of self-control but then consumes an excessively large quantity of the chosen fruit salad? This would be evidence of a lapse in self-control that would be missed by researchers who only observed her choice. Also, if consumers fail to exercise self-control at the choice stage by choosing a vice, will they just devour in the following consumption stage? These questions are relevant to the well-being of consumers because food choice decision and quantity consumption decision jointly determine calorie and nutrient intake. The post-choice consumption stage is usually longer than the choice stage and thus, allows for longer time to decide when to stop eating, as compared to the prior decision of what to eat in the choice stage. However, researchers have rarely considered the possibility that revealed self-control may change across the choice and post-choice consumption stages. In this research, we explicitly test how self-control may change over choice and post-choice quantity decisions within a single consumption episode and find evidence for changing self-control within a consumption episode, highlighting the dynamic operation of self-control.

In what follows, we review literature that has adopted food choice between a vice and a virtue as a measure of self-control and food intake as a measure of self-control. Next, we introduce a theoretical framework which consists of two stages—a choice stage followed by a post-choice consumption stage and discuss how we investigate the potential dynamic of self-control across choice and post-choice consumption stages by examining them together. We then discuss how individual differences can play a role in this two-stage consumption decision framework. Using an endogenous treatment regression model (Krishnamurthi and Raj, 1988) that allows us to analyze quantity contingent on choice, in Study 1, we demonstrate that quantity consumption of a chosen food can reflect divergent levels of self-control depending on individuals’ dietary restraint. When cognitive resources were constrained, among those who chose a virtue, decreasing levels of trait dietary restraint were ironically associated with greater consumption quantities and therefore more calories. This suggests that self-control is no longer sustained among unrestrained eaters after a virtue choice as much as their restrained counterparts. In Study 2, we investigate the underlying process for unrestrained eaters’ losing self-control over their consumption of their chosen virtue, by examining post-choice accessibility of the self-control goal. We conclude with a discussion of theoretical contributions, limitations, and future research.

## 2 Conceptual framework

### 2.1 Self-control and the choice of virtue over vice

Self-control is the “struggle between the two psychological forces of desire and willpower” (Hoch and Loewenstein, 1991). Willpower induces people to act in line with their long-term goals, whereas desire attracts them to temptations that deviate from these goals. Hence self-control is often viewed as the conflict between “sooner-smaller” rewards with “later-larger” outcomes (Ainslie, 1975). Wertenbroch (1998) conceptualized this conflict at the product level with the insight that some products deliver relatively greater value in the short than in the long term, whereas others deliver greater value in the long term. In his terminology, the former class of products are called “relative vices” while the latter class are called “relative virtues.” This distinction between vices and virtues is simple and intuitively appealing, and aligns with consumers’ categorization of foods into good or bad (Okada, 2005; Thomas et al., 2011).

As aforementioned, Shiv and Fedorikhin (1999) operationalized the choice between chocolate cake and fruit salad as a measure of self-control. According to their affective-cognitive model of self-control operation, affective responses favor a vice but cognitive responses need to override affective responses to favor a virtue. Shiv and Fedorikhin demonstrated that when affective processing was facilitated relative to cognitive responses, impulsive (but not non-impulsive) people were more likely to choose chocolate cake than fruit salad. This is how researchers observe choice to make an inference about the decision maker’s self-control enactment: the choice of the vice represents a failure whereas the choice of the virtue indicates a success in enactment of self-control. A pilot survey among lay consumers corroborates researchers’ focus on self-control in the choice stage.^2^

However, such a focus only on choice behavior may provide a possibly incomplete picture of self-control operations since this might change from the choice behavior to the post-choice consumption stage. In order to develop this more complete picture, we first discuss how extant research has corresponded self-control to food consumption quantity.

### 2.2 Consumption quantity and self-control

While much research has investigated self-control as reflected in choice, there has also been an effort to investigate the relationship between self-control and quantity. However, prior research on food intake in relation to self-control has examined consumption while skipping the choice stage (Tice et al., 2001; May and Irmak, 2014; Mehta et al., 2014), limited the available choices to being either only vices or only virtues (Finkelstein and Fishbach, 2010; Redden and Haws, 2013), or investigated yes or no decisions on vices [e.g., whether to eat cookies or not (Ramanathan and Menon, 2006; Coelho Do Vale et al., 2008; Patrick et al., 2009)]. However, consumers often make a food choice first and then decide how much they consume the chosen food. Omitting the choice stage and observing consumption intake only may well have a different impact on self-control compared to observing consumption intake following a free choice. For example, being forced to eat a specific food might lead to a feeling of lack of responsibility (Chen and Sengupta, 2014) or reactance (Finkelstein and Fishbach, 2010), which can potentially influence self-control exertion in the consumption stage. In the current research, as our focus is to assess the dynamics of self-control across the choice decision and the post-choice quantity intake, we avoid forcing such a food type assignment but instead allow choice and quantity decisions to be interdependent.

Examining the manifestation of self-control in post-choice quantity is critical since over-consumption of calories is the single most significant contributor to obesity (Livingston and Zylke, 2012). Inferences of self-control from consumption quantity decisions usually follow a linear relationship, with lower quantities being associated with higher self-control (Aydinoğlu and Krishna, 2011; Belei et al., 2012). Accordingly, in the current research, we treat increasing quantity intake is associated with lower self-control regardless of whether the food is regarded as a virtue or a vice in the choice context. That is because the over-consumption problem is not an exclusive matter of vice foods. Eating only virtuous foods can result in excessive calorie intake and thus consumers need to control the intake of virtues as well as vices.^3^

Importantly, there are no absolute virtues or vices by definition – they are defined relative to each other (Wertenbroch, 1998; Vosgerau et al., 2020), and malleable across contexts (Levin and Gaeth, 1988; Irmak et al., 2011). Many food items used as virtues in experiments (e.g., fruit salad and granola bars) are well above acceptable levels of taste, and the caloric density of these foods is not negligible. Indeed, contrary to stereotypical beliefs, virtues can contain more calories than vices (Oakes and Slotterback, 2005; Howlett et al., 2009), and even stereotypically virtuous foods can have adverse health outcomes if consumed in excess (Ulrich and Potter, 2006). This is consistent with a pilot survey we ran, which showed that lay consumers do understand that virtuous foods should not be overconsumed.^4^

Taken together, it is evident that virtues as well as vices can indeed be over-consumed – in terms of energy and nutrients. One can have too much of a good thing. Consequently, there is a need for self-control to regulate consumption quantity, for vices as well as virtues. However, a limitation in the extant literature is that most studies consider choice and quantity consumption decisions separately. In this research, we aim to extend our investigation to post-choice consumption behavior to see how self-control operates dynamically across choice and quantity decisions.

### 2.3 A two-stage decision framework for self-control operation In food consumption

Food consumption can be analyzed as a two-stage decision process wherein a consumer first chooses what to eat (choice stage), and then decides how much of the chosen option to consume (quantity stage) (Drewnowski, 1997; Wansink and Chandon, 2014). In some decision contexts, food choice and quantity decisions can be made simultaneously [e.g., choosing the flavors and the number of scoops at an ice cream parlor (Oh et al., 2022)], and mixtures of vices and virtues may be chosen [e.g., choosing from vice-virtue bundles with different relative proportions (Liu et al., 2015)], which is beyond the scope of our investigation. As discussed, self-control processes may be relevant to both stages—they may be manifested in both choice and quantity decisions. The untested premise is whether self-control manifested in choice may persist to the post-choice consumption stage. If the chooser’s self-control persists in the post-choice consumption stage, post-choice quantity consumption should follow parallel patterns: those who choose a vice should eat a greater quantity of their chosen item because greater quantities consumed are representative of weaker self-control, whereas, in contrast, those who choose a virtue should eat less. However, if we allow for the possibility that self-control operates dynamically in the two-stage decision framework, a number of different possibilities emerge in the post-choice consumption stage. For example, it is possible that consumers who chose a virtue might end up eating large quantities because they deplete their self-regulatory resources (Muraven and Baumeister, 2000); or they feel it is acceptable to do so (Scott et al., 2008). By contrast, it is also possible that consumers who chose a vice might decide to eat a small quantity of the chosen vice, which can be an example of planned indulgence (Kivetz and Simonson, 2002). Moreover, once a consumer starts eating a chosen food, many factors other than self-control (e.g., hunger, in Nederkoorn et al., 2009) can affect the quantity eaten (Yeomans, 1998; Sclafani, 2001; Wren et al., 2001; Mela, 2006; Wansink and Chandon, 2014), which suggests that one’s self-control exertion can be changed at the consumption stage. Correspondingly, the choice of a vice does not automatically lead to a large quantity eaten; and vice versa. In other words, it is possible that self-control does not always persist over choice and quantity stages. Therefore, for a comprehensive understanding of the entire self-control process within a consumption episode, it is necessary to investigate choice and quantity consumed together.

Surprisingly, with one exception (Fedorikhin and Patrick, 2010), consumer psychologists investigating self-control operations have largely not measured choice and quantity together in a sequence. Fedorikhin and Patrick (2010) found that in a baseline arousal and positive (vs. neutral) mood condition, participants were more likely to choose grapes than M&M’s, and consumed smaller quantities of M&M’s but not grapes once chosen. It is apparent from these findings that self-control manifested in the choice stage can be manifested differently in the consumption stage. Participants were more likely to choose the virtue (high self-control exerted in the choice stage) but once they chose the virtue, they also did not eat less (no longer high self-control in the consumption stage), which is indicative of dynamic self-control operations across the choice and post-choice consumption stages. In particular, self-control expressed in the virtue choice did not necessarily continue to the post-choice consumption stage, in the form of lower intake.

### 2.4 Dynamics of self-control over choice and quantity decisions

While Fedorikhin and Patrick (2010) findings suggest a potential dynamic operation of self-control across choice and quantity consumption decisions, no systematic research has examined how self-control plays out across choice and quantity decisions. Dynamics are inherent in some conceptualizations of self-control. Within an individual, two players conflict: a planner who is far-sighted and thus endorses long-term preferences and a doer who is short-sighted and thus endorses short-term preference (Thaler and Shefrin, 1981; Bénabou and Pycia, 2002). Even when consumers intend to regulate their food intake at the time of making a choice, their will might not necessarily persist till the successful enactment of self-control at the time of post-choice consumption due to the doer’s different preferences from the planner’s. Indeed, a meta-analysis demonstrates that individuals’ trait self-control is related to their imagined self-control behavior to a greater degree than to actual self-control behavior (De Ridder et al., 2012). This suggests that the enacted self-control via choosing a virtue might not be always evinced in moderated consumption behavior once one has started to consume the chosen virtue.

In exploring whether self-control persists across choice and post-choice consumption decisions within individuals, we utilize individual differences in self-control as a basis to distinguish those who are likely to maintain their self-control over decisions (e.g., making a virtue choice in the choice stage and then keeping low food intake in the consumption stage) from those who are likely to lose self-control over decisions (e.g., making a virtue choice in the choice stage but showing high food intake in the consumption stage). Due to the lack of prior investigation that observes post-choice consumption behavior, we adopt an abductive approach that emphasizes theory development based on observation of actual behaviors (Baumeister et al., 2007) followed by mechanism testing (Janiszewski and Van Osselaer, 2022). Hence, for an exploratory investigation, we tested three individual difference measures pertinent to self-control, that can potentially capture the dynamics of self-control over choice and quantity decisions. First, as a measure specific to the food consumption domain, we assessed dietary restraint (Herman and Polivy, 1980). Individuals with high dietary restraint tend to regulate intake of vice food items (Hofmann et al., 2007) and are likely to keep being successful in intake regulation across occasions (van Koningsbruggen et al., 2013). This suggests that those with high dietary restraint, restrained eaters, may be likely to exhibit persistent self-control over choice and quantity decisions, whereas those with low dietary restraint, unrestrained eaters, may be likely to exhibit lost self-control in their post-choice quantity decision stage once they exercise self-control in the choice decision stage. In case of a self-control lapse by choosing a vice in the choice stage, however, it is also an empirical question whether individuals may show the ongoing self-control lapse in the consumption stage. Analogously the same patterns can be predicted with other individual difference measures relevant to self-control, which are more general and less domain-specific, such as trait self-control (Tangney et al., 2004) and consumer impulsivity (Puri, 1996). Those with high self-control or those with low impulsivity may exhibit persistent self-control over choice and quantity decisions (e.g., low food intake after a virtue choice), but those with low self-control or those with high impulsivity may exhibit transient self-control over these decisions (e.g., high food intake after a virtue choice).

### 2.5 Overview of studies

We conducted two studies to explore how self-control operations manifest over choice and quantity decisions. Our empirical strategy for Study 1 referred to Shiv and Fedorikhin (1999) seminal research, conceptually following their design and procedures closely, and then extending these to include consumption quantity as a dependent variable. Specifically, Study 1 tested how (b) post-choice quantity consumption was influenced by choice and individual differences. Study 1 provided initial evidence that self-control changes across the choice stage and the post-choice consumption stage: after having chosen a virtue, under cognitive load, unrestrained eaters consumed greater quantities and therefore more calories, which reflects their lost self-control after exercising it in the choice stage. To investigate the underlying mechanism of the effects observed in Study 1, in Study 2, we examined how food choice and dietary restraint interactively influenced the post-choice accessibility of self-control. We found that after having chosen a virtue, unrestrained eaters showed lower accessibility of a self-control goal, supporting the account based on goal accessibility. These studies together reveal that self-control operation is dynamic across choice and post-choice intake decisions and whether self-control is sustained or lost across depends on individual’s dietary restraint.

## 3 Study

### 3.1 Study 1: divergent inferences of self-control from choice versus quantity

The purpose of Study 1 was to examine how post-choice consumption of a chosen food could be determined by choice behavior and individual differences. Specifically, we tested whether actual consumption would show changes in manifestations of self-control from food choice, and whether this varied by individual difference measures including dietary restraint (Herman and Polivy, 1980) as a measure of domain-specific self-control, general trait self-control (Tangney et al., 2004) and consumer impulsivity (Puri, 1996). We followed the design of Shiv and Fedorikhin (1999) Study 2, aiming to extend their work by measuring post-choice consumption.

According to Shiv and Fedorikhin (1999), individual differences in self-control are manifested in choice behavior under the following conditions: (1) when cognitive resources are limited and (2) affective responses are facilitated (Metcalfe and Mischel, 1999). The first condition was operationalized by imposing high (vs. low) cognitive load, and the second condition by presenting real (vs. symbolic) food options. These conditions were introduced to weaken the executive control but to intensify the effect from affective responses favoring a vice, particularly among those with low trait self-control. Hence, the changing nature of self-control over choice and quantity decisions should better be observed if these conditions are satisfied. Following their implementations, we manipulated cognitive load and also presented participants with the real foods to choose among. Note that we maintained the cognitive load manipulation through the choice phase into the consumption phase. Extending the study from choice to consumption necessarily implies using real foods at the consumption stage, which should evoke affective responses, thereby satisfying the second criterion.

Consistent with Shiv and Fedorikhin (1999), we predicted that imposing high (vs. low) cognitive load should increase choice likelihood for the vice among those with low self-control (e.g., unrestrained eaters). More importantly, we aimed to examine how self-control enactment may change in the post-choice quantity decision, particularly among those low in trait self-control. Specifically, we expected among those who chose a virtue, those with high self-control will consume smaller quantities compared to their counterparts with low self-control, indicating better sustained self-control in the consumption stage. In other words, self-control should be differently evident over the choice and post-choice quantity consumption decisions, depending on individual differences in self-control, thereby revealing the dynamics of self-control within a single consumption episode.

#### 3.1.1 Method

##### 3.1.1.1 Participants and design

Undergraduate students at a major Asian university (*N* = 671, 59.5% female; *M*_age_ = 20.21) participated and were randomly assigned across conditions of a 2 (cognitive load: low vs. high) x 2 (food type: vice vs. virtue) x (individual differences) design, with cognitive load manipulated between-subjects, and food type and individual difference scales (dietary restraint, self-control, and consumer impulsivity) measured. In all studies, all participants provided their informed consent in a written form before the participation. Before collecting data for all studies, the Human Participants Research Panel at Hong Kong University of Science and Technology reviewed and approved the proposed safety measures for the proposal {BM042}. Participants chose between virtue and vice options as described below. Conditional on choice, we assessed the amount that each participant consumed.

##### 3.1.1.2 Pretest and posttest

Following Hagen et al. (2017), we chose almonds and M&M’s as the virtue and vice options in our stimuli. The key consideration was that these are both relatively easy to consume and weigh in discrete units (compared to Shiv and Fedorikhin’s cake and fruit salad, which are relatively more heterogenous in serving sizes and messier to consume and therefore weigh). We conducted one pretest and one posttest to ensure that our participants did indeed perceive these options to be a vice and a virtue as per their definitions. First, in the pretest, 36 participants (72.2% female, *M*_age_ = 20.64) were recruited from the same population pool as the main experiments. Participants saw pictures of the unsalted roasted almonds (private label) and M&M’s, which were contained in transparent plastic cups respectively, and evaluated the healthiness and tastiness of both items on 7-point scales. As expected, paired *t*-test revealed that almonds (*M* = 5.22) were perceived as healthier than M&M’s (*M* = 2.56), *t*(35) = 8.50, *p* < 0.001, but M&M’s (*M* = 6.14) were tastier than almonds (*M* = 4.31), *t*(35) –6.82, *p* < 0.001.

Second, the posttest examined whether people perceived M&M’s contain more calories than almonds, which is consistent with expectations for vice versus virtue foods (Oakes and Slotterback, 2005; Chandon and Wansink, 2007). In the second pretest, 109 undergraduate students (58.7% female) estimated the calories of M&M’s and almonds based on the same pictures as pretest 1. Participants believed the presented quantity of M&M’s (*M* = 437.78 calories), contained higher calories than the almonds (*M* = 294.28 calories), *t*(108) = −6.65, *p* < 0.001. In reality, according to the nutrition labels on the respective packages, roasted almonds (220 calories) contain more calories than M&M’s (196 calories), given the same weight (40 grams). The gap between estimated calories of two options did not depend on dietary restraint, *B* = 1.37, *SE* = 3.77, *t*(107) = 0.36, *p* = 0.717. Thus, we proceeded with M&M’s and almonds as our stimuli.

##### 3.1.1.3 Procedure

Participants came to the lab in groups of one to six, and were met by a research assistant. Each participant was seated in a separate cubicle and worked on an individual PC. Participants read the instructions and responded to a questionnaire programmed on a Qualtrics survey webpage. The experiment was disguised as research on the influence of numeric processing on food tasting. All participants first indicated how hungry they felt at the moment (on a 7-point scale). And then, we manipulated cognitive load by asking participants to remember either an 8-digit number (high cognitive load condition), or a 2-digit number (low cognitive load condition). After this manipulation, we presented all participants with two actual food options (see Supplementary Figure S1), and asked them to choose one to taste. Whichever choice they made, they received 40 grams of their chosen option in a non-transparent plastic cup. These portions had been pre-weighed beforehand in a separate room using an electronic scale. After all participants received their choice of food, they were allowed 4min to taste their chosen option. We then relieved the cognitive load and asked participants to evaluate the taste of their chosen food (4 items, *α* = 0.94) with filler questions, which were unrelated to this study. Then, we administered a series of scales for individual differences in self-control including Herman and Polivy (1980) dietary restraint scale (10-item, *α* = 0.77) as a domain-specific measure of self-control, Puri (1996) consumer impulsiveness scale (11-item, *α* = 0.58,^5^ with one item “eating spending” modified to “enjoy eating” and one item “extravagant” dropped for its low relevance to eating domain), and Tangney et al. (2004) brief self-control scale (13-item, *α* = 0.82).^6^^,^^7^ Finally, demographic questions, including age and gender, were asked. Participants were then debriefed and thanked. After they had all left the lab, a research assistant weighed the amounts left in each participant’s cup. Calorie intake was then estimated using the food choice and quantity eaten. Supplementary Table S1 contains descriptive statistics and correlations of measured variables.

##### 3.1.1.4 Statistical analysis strategy

We have two main dependent variables of interest: food choice and quantity consumed. In analyzing these two variables, we adopted different strategies since food choice is a standalone decision but quantity consumption is conditional on this initial choice. Hence, for the food choice measure, we used a binary logistic regression to test the effect of measured individual differences in self-control and cognitive load on choice. For the post-choice quantity consumption, however, the quantity measure is subject to the issue of self-selection because participants were free to make a choice, not randomly assigned to a specific choice. Put differently, an unobservable factor that is not related to self-control may have influenced choice and quantity simultaneously, suggesting a problem of endogeneity. To address this potential endogeneity, we analyzed quantity consumption using an endogenous treatment regression model (Heckman, 1979; Maddala, 1983) that follows a two-step maximum likelihood estimation. Adoption of this model enables us to analyze the quantity while controlling for a role of unobservable variables that might affect both choice and quantity decisions. This is a common practice in the marketing science literature, analogous to the analysis of purchase quantity conditional on brand choice (Krishnamurthi and Raj, 1988). In a recent consumer psychology application of this method, Galoni and Noseworthy (2015) used it to test whether their participants spent different amounts of money conditional on which aisle of a mock supermarket they chose to shop in (the aisles contained different types of products). To conduct a binary logistic regression, we used the SPSS. To conduct an endogenous treatment regression analysis, we used the *etregress* command in Stata version 17.0. Details of the model specifications will be discussed below.

#### 3.1.2 Results

##### 3.1.2.1 Food choice

Overall, 35% of participants chose almonds while 65% chose M&M’s for tasting. In a binary logistic regression, food choice (0 = “vice”; 1 = “virtue”) was regressed on a cognitive load dummy (0 = “low”; 1 = “high”), dietary restraint (standardized), and their interaction. The regression revealed no significant main effect of dietary restraint, *B* = 0.00, *Wald* = 0.00, *p* = 0.974 (odds ratio = 1.00), but a significant main effect of cognitive load, *B* = −0.41, *Wald* = 6.12, *p* = 0.013 (odds ratio = 0.66), which was qualified by a significant interaction, *B* = 0.45, *Wald* = 7.15, *p* = 0.008 (odds ratio = 1.56). Replacing dietary restraint with the other scale measures caused the interaction to drop from significance, *p*s > 0.55. Follow-up spotlight analyses revealed that the effect of cognitive load was significant at 1 *SD* below the mean of the dietary restraint scale (among unrestrained eaters; *B* = −0.86, *Wald* = 12.24, *p* < 0.001, odds ratio = 0.43), showing that unrestrained eaters were more likely to choose a vice under high (vs. low) cognitive load (*M*_low_ = 60.71% vs. *M*_high_ = 78.41%). In contrast, imposing high (vs. low) cognitive load did not influence choice at 1 *SD* above the mean of the restraint scale (*M*_low_ = 60.52% vs. *M*_high_ = 59.63%; *B* = 0.04, *Wald* = 0.03, *p* = 0.87, odds ratio = 1.04). In addition, simple slopes analyses in the high load condition indicated that decreasing dietary restraint was indeed related to a greater propensity to choose the vice, *B* = 0.45, *Wald* = 12.88, *p* < 0.001 (odds ratio = 1.57). These patterns replicate those reported by Shiv and Fedorikhin (1999). However, we find moderation only by dietary restraint, not by consumer impulsivity or trait self-control. This may possibly be because Shiv and Fedorikhin (1999) did not use the full consumer impulsivity scale developed by Puri (1996) and because the reliability of this scale was unexpectedly low (*α* = 0.58). Also, it is possible that dietary restraint is a more specific measure of self-control in the food consumption domain than the others.

##### 3.1.2.2 Quantity consumed in grams

Endogenous treatment regression estimates a set of predictors for food choice, and a set of predictors for quantity consumed together, and tests whether there is indeed a significant endogeneity due to choice being self-selected. In the equation for choice, cognitive load, dietary restraint and their interaction were used as predictors, as we analyzed the choice measure, while in the equation for quantity consumed, cognitive load, choice, dietary restraint, and all two- and three-way interactions between these variables were used, controlling for subjective hunger and the taste of sampled food (see Supplementary Table S2 for the details). Control variables were introduced due to their potential impact on intake (Guerrieri et al., 2008; Nederkoorn et al., 2009).

**Figure 1:**
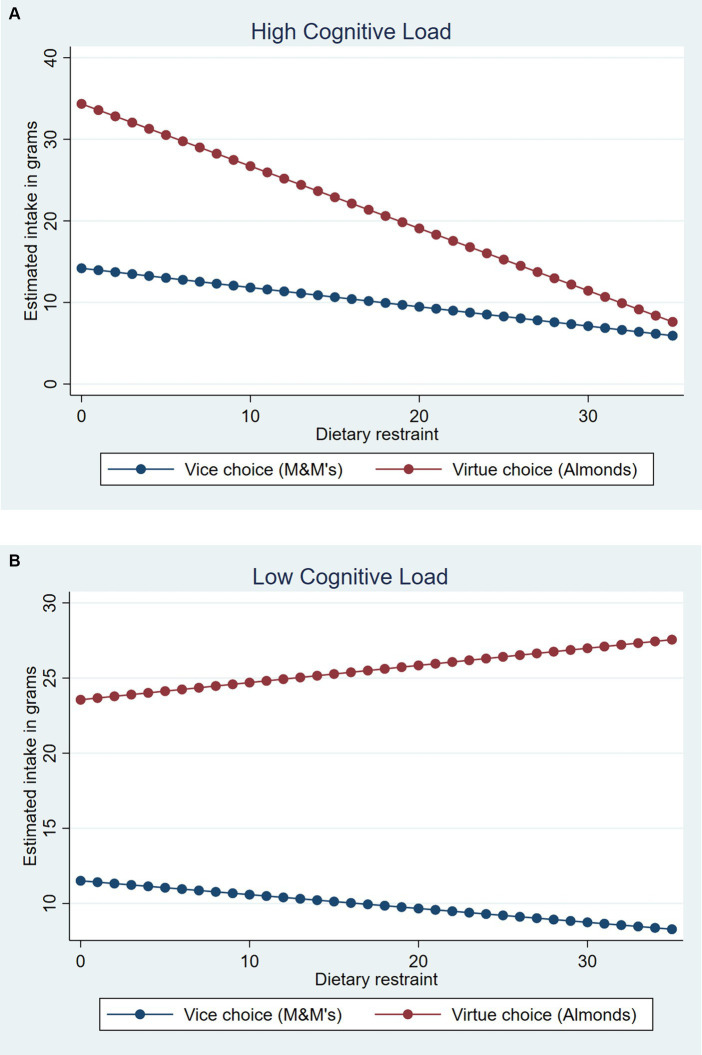
Quantity consumed in grams as a function of cognitive load, food choice, and dietary restraint in study 1. **(A)** High cognitive load. **(B)** Low cognitive load. Estimates plotted based on raw dietary restraint scores (range: 0–35).

There was a significant endogeneity between food choice and quantity in this case (*ρ* = −0.76, *σ* = 11.21, *χ^2^*(1) = 44.82, *p* < 0.001), highlighting the value of using this method. Specifically, controlling for subjective hunger, *B* = 1.60, *z* = 8.30, *p* < 0.001, and taste of the sampled food, *B* = 1.01, *z* = 3.07, *p* = 0.002, the regression revealed a significant main effect of choice, *B* = 14.68, *z* = 6.89, *p* < 0.001, and a significant three-way interaction, *B* = −4.01, *z* = −2.66, *p* = 0.008. No other main effects or interactions reached significance (*p*s > 0.29). We conducted simple slopes analyses in order to test the effect of dietary restraint conditional on choice in each cognitive load condition. Critically, in the high cognitive load condition, after having chosen a virtue, increasing dietary restraint was associated with lower quantities consumed, *B* = −4.17, *z* = −4.31, *p* < 0.001 (see Figure 1). This suggests that despite self-control enactment in the choice stage, those with low dietary restraint rather end up losing self-control in the consumption stage while those with high dietary restraint rather maintain their self-control in the consumption stage. In contrast, after having chosen a vice under high cognitive load, increasing dietary restraint was not significantly associated with lower quantities consumed, *B* = −1.29, *z* = −1.80, *p* = 0.071 (but see the results on the estimated calorie consumption below). In the low cognitive load condition, dietary restraint did not affect quantity consumed regardless of the chosen option, *p*s > 0.51. Finally, when we replaced the dietary restraint scale with the other general individual difference measures (consumer impulsivity and brief self-control scales, respectively), none of the three-way interactions were significant (*p*s > 0.80). Finally, we conducted parallel analyses using OLS that does not account for endogeneity, and obtained similar results in terms of patterns and significance levels (see Supplementary Table S3 for details).^8^

##### 3.1.2.3 Calorie intake

We analyzed calorific intake using the same endogenous treatment regression model with the same set of predictor equations. While there was no significant endogeneity here, *ρ* = −0.54, *σ* = 51.33, *χ^2^*(1) = 2.44, *p* = 0.12, we accounted for endogeneity in the subsequent analyses (see Supplementary Table S4). The regression revealed a significant main effect of choice, *B* = 59.40, *z* = 2.15, *p* = 0.03, and the significant target three-way interaction, *B* = −19.66, *z* = −2.46, *p* = 0.014, controlling for subjective hunger, *B* = 8.30, *z* = 8.25, *p* < 0.001, and taste of the sampled food, *B* = 5.07, *z* = 2.92, *p* = 0.003. No other effects were significant (*p*s > 0.35). We again conducted simple slopes analyses within each choice x load condition. Under high cognitive load, after having chosen a virtue, increasing dietary restraint was associated with lower calorie consumption, *B* = −19.13, *z* = −3.30, *p* = 0.001 (see Supplementary Figure S2). However, after having chosen a vice under cognitive load, dietary restraint did not affect calorie intake, *B* = −4.58, *z* = −1.13, *p* > 0.257. Under low cognitive load, dietary restraint did not influence calorific intake for either virtue or vice choice, *p*s > 0.52. Again, the other trait measures did not produce any meaningful results (3-way interactions, *p*s > 0.82). Note that we also conducted parallel analyses using OLS that does not account for endogeneity, and obtained similar results in terms of the patterns and significance levels (Supplementary Table S5).

#### 3.1.3 Discussion

These results demonstrate that self-control can change over choice and quantity decisions, depending on individuals’ dietary restraint. Similar to Shiv and Fedorikhin (1999), in Study 1, we found a significant interactive effect of cognitive load by dietary restraint on choice of vice vs. virtue. Unrestrained eaters were far more likely to choose a vice when under cognitive load; load had no effect for restrained eaters. The behavior of individuals who have low self-control (i.e., unrestrained eaters) is well captured by the choice measure under high cognitive load. This is a conceptual replication of Shiv and Fedorikhin’s (1999) findings. Furthermore, analysis of quantity consumed tells a novel story of dynamic self-control. In the high cognitive load condition, among those who chose a virtue, decreasing dietary restraint was associated with increasing quantities consumed and higher calorific intake. In other words, when unrestrained eaters successfully enacted self-control in the choice stage by choosing the virtue, they exhibited their lack of self-control over actual consumption by increasing intake of the chosen virtue. In contrast, when participants chose a vice, quantity consumed was not dependent on their dietary restraint. Study 1 therefore provides evidence that self-control may change across choice and quantity decisions within a single consumption episode. Also, such a dynamic is clearly captured when unrestrained eaters compare to restrained eaters, but not captured when individual differences are accounted based on general trait self-control or impulsivity.

Why might this happen? From the observed patterns, we propose that choice behavior itself in the first stage could influence self-control goal accessibility, depending on dietary restraint. Specifically, for people with low levels of dietary restraint, merely choosing a virtue would decrease accessibility of the self-control goal (Shah, 2005), leading to higher quantity consumption. Such an effect would not be observed among people with high dietary restraint. To investigate this possible mechanism, in Study 2 we examined whether goal accessibility might play a role across choice and quantity decisions by measuring post-choice goal accessibility.

### 3.2 Study 2: post-choice accessibility of the self-control goal

Prior research on goal pursuit across multiple decisions has shown that sufficient progress on a self-control goal due to a prior decision or behavior can lower the activation of the self-control goal, and increase the activation of a conflicting goal (Fishbach and Dhar, 2005; Shah, 2005; Laran and Janiszewski, 2009). For example, when a past instance of restraint is salient, people low in self-control (e.g., impulsive consumers) tend to indulge themselves, and this is due to lowered accessibility of the self-control goal (Mukhopadhyay et al., 2008). Applying this goal accessibility account to the current instance, the act of choosing a virtue in the choice stage might decrease the accessibility of the self-control goal for unrestrained eaters, presumably due to their weak interest in controlling food decisions—thereby causing them to eat greater quantities in the consumption stage. In contrast, the same virtue choice should not have this effect for restrained eaters, but rather they can sustain their self-control in the post-choice consumption stage after making a virtuous choice because they by definition are motivated to regulate food consumption and have a chronically high goal of self-control in this domain. Hence, we predict that after choosing a virtue, more unrestrained eaters should respond more slowly to self-control related words in a lexical decision task.

#### 3.2.1 Method

##### 3.2.1.1 Participants and design

Undergraduate students (*N* = 356, 54.8% female; *M*_age_ = 20.02) participated in exchange for course credit. As in Study 1, participants freely chose between a vice (M&M’s) and a virtue (almonds), and their dietary restraint (*α* = 0.77) was measured.

It is worth noting that in Study 1 we observed our focal effects under high cognitive load, but in this study, we did not limit processing resources. This is because activation of self-control goals (as opposed to behaviors) is not affected by availability of cognitive resources. Prior research found no difference in accessibility of goals that are relevant to self-control between high and low cognitive load conditions (Fishbach et al., 2003). Hence, to avoid further complicating the already complex procedure, we did not constrain processing resources in this Study.

##### 3.2.1.2 Procedure

This study was presented as a study on visual processing and taste perception. Participants were run in groups of up to nine at a time. All participants were seated at individual workstations, and first chose a food and then performed a lexical decision task. The procedure for the food choice was similar to Study 1, but with some important differences. Each participant was presented with almonds and M&M’s, side by side in separate transparent plastic cups (see Supplementary Figure S3). The cups were sealed with a transparent lid to prevent participants from taking and tasting any. After everyone had indicated their choice, the sealed bowl containing the chosen option was placed right below the computer screen, directly in front of the participants, who were asked to proceed to the lexical decision task (see Supplementary Figure S4 for the setting for this task).

The lexical decision task (conducted on DirectRT version 2010.2) was disguised as a visual processing task. Participants were instructed to identify whether the letter string on the screen was a word or a non-word by pressing the yellow key for a word (yellow dot sticker on key C) or the green key for a non-word (green dot sticker on key N). They were instructed to respond as accurately and fast as possible. After 5 practice trials, they did 40 trials, of which 5 words were related to self-control (fit, health, diet, weight, and slim), 5 were related to indulgence (delicious, indulge, eat, yummy, tasty), 10 words were neutral (balloon, desk, folder, picture, shoe, printer, sink, pen, card, wall) and there were 20 non-words (Wilcox et al., 2009; Fedorikhin and Patrick, 2010; Laran, 2010a; Laran, 2010b). The presentation order of trials was randomized. In each trial, the fixation point (+) was presented for 1,000 milliseconds, and was followed by a target.

Following the lexical decision task, the experimenter informed participants that the tasting task was cancelled due to time constraints, and they had to continue to the surveys on their PC. The experimenter collected the food cups from each participant while ensuring that no one had consumed any during the study. Participants then worked on filler tasks for around 20 min, then responded to dietary restraint scale (10-item, *α* = 0.77), consumer impulsivity scale (11-item, *α* = 0.63), and brief self-control scale (13-item, *α* = 0.82) as in Study 1.^9^ Descriptive statistics and correlations among measured variables are reported in Supplementary Table S6.^10^

##### 3.2.1.3 Statistical analysis strategy

This study has two types of dependent variables of interest: (1) food choice and (2) goal accessibility measures for a self-control goal and an indulgence goal. For the food choice measure, we adopted the same analysis strategy as in Study 1, using binary logistic regression (this study did not feature a cognitive load factor). For the goal accessibility measures (facilitation scores), since food choice was self-selected as before, we adopted endogenous treatment regression to analyze each measure separately. The same statistical software was used for these analyses as in the Study 1. Details of the model specifications are discussed below.

#### 3.2.2 Results

##### 3.2.2.1 Food choice

Similar to Study 1, 30.6% of participants chose the virtue and 69.4% chose the vice. In a binary logistic regression, we regressed choice (0 = “vice”; 1 = “virtue”) on dietary restraint. As we did not manipulate cognitive load in this study, dietary restraint did not influence choice likelihood, *B* = −0.15; *Wald* = 1.63; *p* = 0.20 (odds ratio = 1.16), consistent with the low cognitive load condition of Study 1.

##### 3.2.2.2 Facilitation scores from response latencies

Before analyzing response latencies, the data were prepared by dropping false responses (3.6% of all responses) that misidentified the targets, due to difficulty in interpreting such incorrect responses (Bargh et al., 1992). Further, we excluded latencies that were faster than 300 milliseconds or slower than 2000 milliseconds (0.5% of all responses), following prior practices (Anderson et al., 1998; Leibold and Mcconnell, 2004; Mukhopadhyay et al., 2008). As a measure of relative accessibility of a target goal compared to neutral words, we constructed facilitation scores by subtracting the average response time for words of the target category from the average response time for neutral words (Anderson et al., 1998; Leibold and Mcconnell, 2004; Förster et al., 2005; Finkelstein and Fishbach, 2010). Greater facilitation scores mean faster responses to the target category, suggesting higher accessibility of the target goal. Two facilitation scores were constructed for each participant: one for the self-control goal, the other for the indulgence goal.

**Figure 2:**
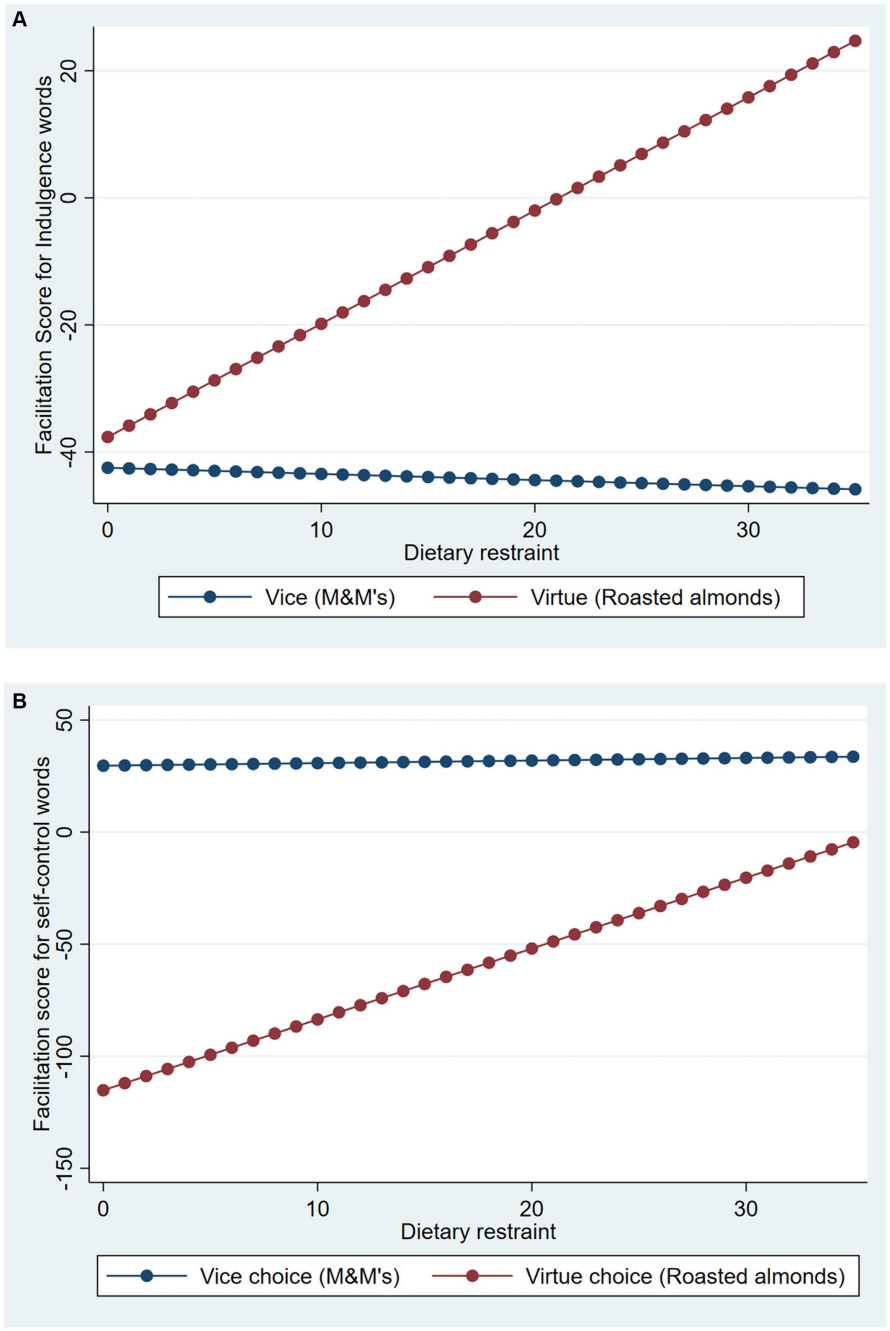
Facilitation scores for self-control goal and indulgence goal as a function of food choice and dietary restraint in study 2. **(A)** Self-control goal facilitation score. **(B)** Indulgence goal facilitation score. Estimates plotted based on raw dietary restraint scores (range: 0–35).

We conducted separate endogenous regression analyses for the two different facilitation scores. For choice, dietary restraint was the predictor and for facilitation scores, the target facilitation score was regressed on choice, dietary restraint, and their interaction. For the facilitation score for self-control, the analysis revealed significant endogeneity, *ρ* = 0.75, *σ* = 84.30, *χ^2^*(1) = 0.11.41, *p* = 0.001 (see Supplementary Table S7). Accounting for this, there was a significant main effect of choice, *B* = −106.61, *z* = −3.79, *p* < 0.001, an insignificant effect of dietary restraint, *B* = 0.64, *z* = 0.15, *p* = 0.88, and a significant interaction, *B* = 16.85, *z* = 2.41, *p* = 0.016. Simple slopes analyses revealed that when the vice was chosen, dietary restraint did not affect facilitation of self-control, *B* = 0.64, *z* = 0.15, *p* = 0.88 (see Figure 2A). However, when the virtue was chosen, dietary restraint significantly affected self-control facilitation, *B* = 17.49, *z* = 2.92, *p* = 0.004. This supports our prediction in that after virtue choice, participants with low dietary restraint responded slower to words related to self-control compared to those with high dietary restraint—self-control was less accessible for them.

Analysis of the facilitation score for the indulgence goal revealed no significant endogeneity, *ρ* = −0.18, *σ* = 85.65, *χ^2^*(1) = 0.91, *p* = 0.341 (see Supplementary Table S8). Moreover, no effects were significant (choice, *B* = 28.36, *z* = 1.00, *p* = 0.32, dietary restraint, *B* = −0.53, *z* = −0.10, *p* = 0.92, interaction, *B* = 10.39, *z* = 1.30, *p* = 0.19) (see Figure 2B). All the above patterns remained similar when analyses were conducted using standard OLS (see Supplementary Table S9). Additionally, replacing dietary restraint with the other trait measures reproduced none of the above results (i.e., *p*s of choice x trait scales >0.3).

#### 3.2.3 Discussion

These results provide strong and convergent support for our propositions based on goal accessibility account for the dynamic of self-control. After having chosen a virtue, unrestrained eaters showed decreased accessibility of words related to self-control, supporting our goal-accessibility based prediction. However, for restrained eaters, even after having chosen a virtue, a self-control goal remained highly accessible. We can infer that this difference in goal accessibility would have contributed to the observed differences in the post-choice quantity consumption (Belei et al., 2012). In Study 1, unrestrained eaters who chose a virtue may have satisfied their self-control goal by their choice behavior, leading to a backfire effect as evidenced by increased consumption of the chosen virtue. In contrast, among those who chose a vice, dietary restraint did not affect the accessibility of self-control. This result is consistent with the quantity patterns we observed in Study 1, where we found no effect of dietary restraint after a vice choice.

Why did we not observe any significant effects for the accessibility of indulgence? It is possible that the choice context itself, consisting of a vice and a virtue presented side by side, can suppress activation of the indulgence goal regardless of one’s decision. This is consistent with Fedorikhin and Patrick (2010) observation that when actual vice and virtue options (M&M’s and grapes) were presented (vs. not), the accessibility of self-control was increased but that of indulgence was decreased.

## 4 General discussion

By examining choice and post-choice quantity together, this research highlights the benefit of observing post-choice consumption behavior following choices to understand the dynamic of self-control within a consumption episode. After successful enactment of self-control by making a virtuous choice, unrestrained eaters ate greater quantities and consequently more calories compared to their restrained counterparts, if their processing resources were limited (Study 1). While successfully choosing a virtue is one indication of high self-control, in the subsequent quantity decision stage, their self-control is not always sustained in the post-choice consumption stage. Particularly, dietary restraint determined persistence of self-control exercise during the post-choice consumption stage among people who chose a virtue. This shows changes in self-control exercise across choice and consumption stages. In contrast, among those who chose a vice, dietary restraint did not systematically influence consumption quantity. This suggests that although those who chose a vice failed to enact self-control in the choice stage, they did not end up eating too much of the chosen vice. We further examined the underlying mechanism for this effect by measuring post-choice goal accessibility (Study 2). After having chosen a virtue, decreasing dietary restraint was associated with lower accessibility of the self-control goal, suggesting that the choice of a virtue deactivates self-control thereby increasing consumption quantity among unrestrained eaters. Together, the result suggests that in the common consumption context where consumers make a food choice first and then decide how much to consume the chosen food, self-control can operate dynamically across choice and quantity decisions, depending on individuals’ motivation in dietary regulation. Notably, while self-control in the consumption stage is lost for unrestrained eaters when they successfully exercised self-control in the choice stage, such a lapse in the consumption stage is not similarly observed when they failed to exert self-control in the choice stage. Presumably, because the post-choice consumption stage usually allows for a longer duration for the consumption decision, those who initially exhibited a self-control lapse by choosing a vice, regardless of their dietary restraint, might be better able to recover from their self-control lapse by taking time to correct their decision and moderate their intake of the chosen vice.

Note that in our experimental settings in both studies, participants were instructed to make food consumption decisions to taste, which might be different from decisions to consume in general. Hence, to test whether our design is susceptible to such a problem, we conducted a post-hoc test (*N* = 201, 50.2% female, *M*_age_ = 40.53; American participants recruited from Amazon Mechanical Turk) in which we asked two questions that only differ in the “consume”/“taste” wording in the questions: “Imagine you have a choice between M&M’s and almonds for consumption/tasting. If you chose to consume/taste M&M’s instead of almonds, how much would you think it is reflective of your self-control?” (1 = very low self-control; 7 = very high self-control). The two questions were counterbalanced. Paired samples *t*-test revealed that participants did not give different ratings between the consumption decision (*M* = 3.54) and the tasting decision (*M* = 3.67; *t*(200) = 1.68, *p* = 0.095). Also, notably, we separately ran a one-sample *t*-test to check whether the rating was below the mid-point, which would suggest that the decision to consume/taste M&M’s over almonds is indicative of low self-control. Indeed, the results support the idea that both decisions, for consumption (*t*(200) = −3.94, *p* < 0.001) and for tasting (*t*(200) = −2.88, *p* = 0.004), were perceived as relatively low self-control. The results ensure the generalizability of our findings to consumption.

### 4.1 Theoretical contributions

Shiv and Fedorikhin (1999) seminal research triggered a wave of research in self-control, much of which was based on one of their key propositions, namely, that self-control enactment is manifested in the behavior of choosing a virtue over a vice. However, whether self-control operation might change in the post-choice stage has remained unanswered. Our two-stage analysis demonstrates that the operation of self-control is dynamic across food choice and quantity decisions within a single consumption episode. Post-choice quantity decisions are a critical and integral part of food consumption where the self-control enactment can help avoid excessive consumption and thus support long-term goals. Our two-stage analysis reveals changes in the accessibility of the self-control goal as the reason why restrained eaters maintain self-control over intake of a virtue whereas unrestrained eaters fail to maintain self-control once they start to consume their choice. Prior research has found that exerting self-control, such as by choosing a virtue over a vice, can influence subsequent behavior (Baumeister et al., 1998; Dewall et al., 2007; Gal and Liu, 2011). Other researchers have addressed the question of goal accessibility across consumption episodes (Mukhopadhyay et al., 2008; May and Irmak, 2014). However, no research has looked at how the very act of choosing a virtue over a vice affects the accessibility of the self-control goal within a same consumption episode and governs whether self-control persists or gets lost in the post-choice intake stage. We demonstrate that the choice of virtue (vs. vice) interacts with dietary restraint to influence the accessibility of self-control, thereby explaining the patterns of changing self-control observed across choice and post-choice consumption decisions. These findings map the dynamics of the self-control goal across the choice and consumption stages of a single consumption episode, pronounced among those with weak interest in dietary control.

Based on the goal accessibility account, our finding suggests that unrestrained eaters might perceive their virtue choice as progress towards a self-control goal (Fishbach and Dhar, 2005; Mukhopadhyay et al., 2008; Laran, 2010a). Presumably, this perception of progress caused by the act of choosing a virtue leads unrestrained eaters to relax control over how much they eat (Louro et al., 2007), or to take their choice as a license for consuming more quantity (Khan and Dhar, 2006; Mukhopadhyay and Johar, 2009). Restrained eaters, in contrast, maintain the accessibility of self-control even after a virtue choice, which makes them sustain their self-control without over-consuming. While we find evidence for this goal-accessibility-based mechanism, the patterns of behaviors we observe are consistent with other mechanisms. For example, unrestrained eaters might have consumed larger quantities of the chosen virtue under high cognitive load because they perceived its taste to be better (Van Der Wal and Van Dillen, 2013), perceived large serving sizes to be more appropriate (Provencher et al., 2009), underestimated the calorie content of the virtue (Chandon and Wansink, 2007), or perceived virtuous foods as being light (Deng and Kahn, 2011). Possibly, several mechanisms may work simultaneously. Nonetheless, we found over-consumption of the chosen virtue only in high cognitive load conditions, which may make it difficult for consumers to generate further inferences during their consumption. Thus, other mechanisms that rely on further inferences (e.g., appropriate size; calorie estimation) might be less likely to contribute to unrestrained eaters’ increased virtue consumption.

A third contribution of this research is in introducing endogenous treatment regression models to the self-control literature. As mentioned, these models are fundamental to the marketing science literature, where they were introduced to answer the question, given a consumer chooses a given brand, how much does s/he buy? For example, price discounts may induce brand switching and/or increased purchased quantity, therefore it is important to understand both effects jointly. Similarly, in the consumer psychology literature, a 2-stage estimation model is used to account for self-selection to investigate whether people who had been randomly given either clean new bills or dirty, crumpled, money chose to spend that money on cleaning products or office supplies, and how much they then spent (Galoni and Noseworthy, 2015). Our research follows a very similar estimation method, and we hope that other consumer psychologists and self-control researchers will adopt similar models which have been designed to address questions of this nature which is inevitable in the design.

### 4.2 Trait measures related to self-control

Why did dietary restraint have an effect on quantity consumed and goal accessibility across our studies, but never the other measures of trait self-control? There are several possibilities. The most straightforward is that a domain-specific measure of self-control is more predictive of behaviors than general self-control scales (Haws et al., 2016). Self-control operations are domain-specific (Metcalfe and Mischel, 1999), and it may simply be the case that the general scales we used were not sensitive enough to capture the effects, particularly on post-choice quantity.

Over and above measurement issues, domain-specificity is also implicated if one were to try and understand our observed patterns in terms of motivation rather than ability to self-control. Self-control motivation can lead to internal conflict which increases resistance to temptations (Hofmann et al., 2012), but the outcome of high motivation is not always high self-control behaviors. In contrast, the outcome of high ability to self-control is, by definition, increased restraint. In this research, we operationalized domain-specific self-control motivation as dietary restraint. This is because restrained eaters are known to have a strong conflict between their desires for tasty foods and a chronic goal to restrict their diet (Stroebe et al., 2013; Van Der Laan et al., 2014). Indeed, restrained eaters exhibit stronger resistance against tempting foods (Hofmann et al., 2014). As unrestrained eaters lack such a motivation to control food intake, their self-control operates in a less persistent manner across choice and quantity decision stages than restrained eaters, particularly once they have already exercise self-control in the choice stage.

Additionally, a difference between restrained eaters’ and unrestrained eaters’ exercise of self-control over choice and quantity decisions can be considered as a difference arising from self-control exercised through resolve versus suppression (Ainslie, 2021). Resolve enables more enduring and persistent self-control exercise success compared to suppression that helps exercise of self-control with effort and thus, is difficult to be sustained. Restrained eaters’ chronic motivation to control their intake might help them become adept at exercising self-control without much effort via resolve whereas unrestrained eaters, lacking ongoing motivation to persist dietary regulation, might only exercise self-control via suppression with a great deal of effort. As a result of differing effort levels required for self-control enactment in the choice stage for a virtuous choice, their trajectories of self-control persistence might start to diverge from the post-choice stage.

## 5 Limitations and future research

The current research has several limitations. First, due to the time constraints in the lab, participants were only allowed to consume their chosen food for a predetermined limited time. They may well have eaten more if given more time – although that argument applies across all conditions. Second, for purposes of control and tractability, we adopted the same stimuli across studies, with M&M’s and almonds representing vices and virtues, respectively. Future research should check whether the dynamic operation of self-control across choice and quantity decisions is robust across different foods.

Second, the different tastes of vices and virtues (e.g., sweet, fat, and salty tastes) may impact food liking and satiety differently (Drewnowski and Schwartz, 1990; Bolhuis et al., 2018), and such inherent differences are unavoidable in the current research design. We try to address this issue partially by controlling for the taste of the sample food as rated by individual participants. Also, as food intake is influenced by numerous transitory factors such as hunger level and food variety in the environment (Guerrieri et al., 2008; Nederkoorn et al., 2009), we also controlled for subjective hunger level of participants in Study 1. However, this cannot completely rule out the potential impact of unmeasured transitory factors on food decisions in the current studies.

There is also a question of domain-specificity of dynamic operation of self-control. To understand the operation of self-control, our studies were conducted in the domain of food, but we believe the implications of our results are not restricted to food alone. Food has been the modal domain in self-control research, but similar choice measures have been used to examine dynamic self-control operation in other product categories. For example, Milkman et al. (2009) studied rentals of educational versus entertaining videos, and Mukhopadhyay and Yeung (2010) used the same category in two experiments to assess parents’ and adult caregivers’ choices for children. While documentaries versus action flicks certainly satisfy the criteria for serving as relative virtues versus vices, we believe that binge-watching the History Channel might well be too much of a “good” thing and individuals’ self-control relevant to the entertainment domain may underlie such a case of lost self-control in the post-choice stage. Excessive virtuous behaviors might be damaging because prolonged delay of gratification (e.g., nonstop work without leisure) can harm wellbeing (Grant and Schwartz, 2011). As a more extreme example, hand-washing is a virtuous behavior because it reduces the risk of infection, but compulsive hand-washing is a manifestation of obsessive-compulsive disorder (Hinds et al., 2012). Whether post-choice consumption “quantity” measures follow similar patterns in other domains is an empirical question we leave for future research.

The dynamic operation of self-control across choice and quantity decisions was observed only under high cognitive load. This suggests that the self-control process that drives eating is relatively unconscious, but our results do not definitively indicate when and how consumers decide their intake quantities. As we proposed, the goal accessibility that is influenced by an act of choice can play a role in quantity decisions. Furthermore, however, once consumers start eating, it is possible that external influences such as sensory stimulations from eating (e.g., Drewnowski and Schwartz, 1990) might override initial intentions to control consumption. Since both unconscious and conscious processes can affect self-control over consumption decisions (Williams and Poehlman, 2017), future research should explore how post-choice consumption is shaped by multiple processes that vary in consciousness.

Finally, one may criticize our fundamental analysis strategy on the grounds that observing quantity consumption contingent on choice is susceptible to problems of self-selection. In response, it is important to note that our analyses are always conducted *within* a chosen option – given a choice of vice or virtue, we find differences in quantities consumed based on dietary restraint and cognitive load. More generally, as we have stated, this “limitation” is a feature of the research question, not a bug. The endogenous treatment regression model we use has been developed for this very purpose. Such models are fundamental to marketing science for the last three decades, and have been in use in econometrics for even longer (Heckman, 1979). Indeed, the model we use is primitive enough that it is available in a commonly used statistical software package. Our results show that self-selection is not always a problem in such cases, and when it is, it can be accounted for statistically.

## 6 Conclusion

This research highlights the dynamic nature of self-control over food choice and post-choice consumption decision stages within a single consumption episode. When processing resources were constrained, whether participants continued successful self-control enactment after their initial choice, depended on their dietary restraint. Among those who exhibited self-control in their virtue choice, decreasing dietary restraint was associated with increasing consumption quantities and consequently higher calorific intake. Also, those who exhibited a self-control lapse as evidenced by their vice choice showed moderated intake of the chosen vice, regardless of their dietary restraint, indicating that lost-self-control in the choice stage does not always lead to continued self-control lapse in the consumption stage. Together, these results suggest that the operation of self-control can be dynamic across choice and quantity decisions. This changing self-control within a consumption episode has been neglected in the extant literature due to less investigation of the post-choice consumption stage. Also, we demonstrate that the accessibility of the self-control goal at the post-choice stage contributes to the changes in self-control over choice and quantity decisions.

A true understanding of self-control must consider its dynamics over choice and subsequent quantity decisions.

## Data availability statement

The raw data supporting the conclusions of this article will be made available by the authors, without undue reservation.

## Ethics statement

The studies involving humans were approved by the Human Participants Research Panel at Hong Kong University of Science and Technology. The studies were conducted in accordance with the local legislation and institutional requirements. The participants provided their written informed consent to participate in this study.

## Author contributions

G-EO and AM contributed to the conceptualization, designing the studies, and revisions of the manuscript. G-EO carried out the data collection with research assistants and performed the data analysis under the supervision of AM. G-EO drafted the manuscript and AM contributed to the revisions. Both authors approved the submitted version.

## Footnotes

^1^While the chocolate cake and fruit salad pairing has remained popular (Usta and Häubl, 2011; Biswas et al., 2014), many other sets of stimuli have been used, such that the options selected as vices are perceived to be tastier but less healthy than the ones selected as virtues [e.g., a chocolate bar and a granola bar (Patrick and Hagtvedt, 2012); M&M’s and grapes (Fedorikhin and Patrick, 2010); French fries and salad (Wilcox et al., 2011)].

^2^We conducted a survey to see if people primarily rely on food choice rather than quantity decision for typical food consumption decisions: meal and snack. We recruited American participants (*N* = 200, 43.5% female; *M*_age_ = 43.03) from Amazon Mechanical Turk and asked which one they focus more on between “what I should eat” and “how much I should eat” for a meal and for a snack separately (1 = I focus mainly on “what I should eat”; 7 = I focus mainly on “how much I should eat”). A one-sample t-test revealed that for the meal consumption, people tend to focus more on what they should eat (*M* = 3.32; *t*(199) = −5.12, *p* < 0.001); but for the snack consumption, people equally focus on what they should eat and how much they should eat (*M* = 3.81; *t*(199) = −1.41, *p* = 0.159). Given that meals are more frequent food decisions than snacks and constitute a majority of daily calorific intake (Kant, 2018), this suggests that consumers primarily focus on food choice rather than quantity.

^3^While there is a consensus that over-consumption of vices is a self-control problem, there is a question about the over-consumption of virtues. On one hand, virtues are, by nomenclature, “good,” and hence the more they are consumed, the better. Following such a logic, consequently, the greater the consumption of virtues, the higher the self-control is assumed (Redden and Haws, 2013; Siddiqui et al., 2017). However, this philosophy focuses on the choice part of the equation. The other position focuses more on quantity, specifically, the over-consumption aspect. This position argues that not only are vices best consumed in moderation, but the same should apply to virtues even though the specific reasons may differ. We follow the latter, for the following reasons. Although consumers tend to easily justify eating or purchasing large quantities of virtues (Wertenbroch, 1998; Provencher et al., 2009; Mishra and Mishra, 2011), eating larger quantities necessarily increases calorie intake. Indeed, quantity (not choice), accounts for two-thirds of the variance in energy intake from food consumption (Schusdziarra et al., 2010). Increasing calorific intake can be pleasurable because of increased dopamine release (De Araujo et al., 2008), and this pleasure can induce over-consumption – indicating that self-control may be required, to some extent, to limit consumption of all palatable foods, not just stereotypical vices.

^4^We conducted a pilot survey to examine whether lay consumers also believe that over-consumption of virtues can harm their long-term goal of health, and the results demonstrate that they do. From the same student population pool as in the main studies, 193 undergraduate students (52.3% female; *M*_age_ = 20.17) indicated the extent to which they agreed with each of two statements (1 = I strongly disagree; 7 = I strongly agree): “If some food is good for health, it is okay to eat the food without limiting the quantity” and “Eating a large quantity of healthy foods is always good for health.” The mean belief score (2-item, *α* = 0.76, *M* = 3.38, SD = 1.37) was significantly lower than the mid-point of the scale (target value = 4, one sample t test, *t*(192) = −6.32, *p* < 0.001), indicating that participants generally believed that eating virtues without limiting quantity is not necessarily good for health.

^5^Note that the reliability for the consumer impulsivity scale was unexpectedly low (*α* = 0.58).

^6^For example, the dietary restraint scale includes items such as “How often are you dieting? (0 = never, 1 = rarely, 2 = sometime, 3 = often, 4 = always)”; the consumer impulsivity scale includes “impulsive” (1 = usually would describe me; 7 = seldom would describe me); and the brief self-control scale includes “I am good at resisting temptation (1 = not at all like me; 5 = very much like me).

^7^Factor analysis conducted on each of the scales, using Maximum Likelihood extraction and Varimax rotation, supported the structures of the scales that have been well established in the literature. For the 10-item dietary restraint scale, Barlett’s test of sphericity was significant (χ^2^(45) = 1426.55, *p* < 0.001) and the two-factor structure was revealed (goodness-of-fit χ^2^(26) = 77.54, *p* < 0.001): Factor 1 (Eigenvalue = 3.30) explains 33.01% of variance and factor 2 (Eigenvalue = 1.49) does 14.89%. This is consistent with the literature that has conceptualized and shown two subfactors, namely, concern for dieting and weight fluctuation (Polivy et al., 1978; Heatherton et al., 1988). For the 11-item consumer impulsivity scale, Barlett’s test of sphericity was significant (χ^2^(55) =, *p* < 0.001) and the two-factor structure was revealed (goodness-of-fit χ^2^(34) = 235.60, *p* < 0.001): Factor 1 (Eigenvalue = 4.05) explains 32.84% of variance and factor 2 (Eigenvalue = 1.78) does 16.19%. This is consistent with the literature that has conceptualized and shown two subscales, namely, prudent and hedonic (Puri, 1996). Lastly, for the 13-item brief self-control scale, Barlett’s test of sphericity was significant (χ^2^(78) = 1997.01, *p* < 0.001) and a three-factor structure emerged (goodness-of-fit χ^2^(42) = 127.60, *p* < 0.001): Factor 1 (Eigenvalue = 4.12) explains 31.71% of variance, factor 2 (Eigenvalue = 1.38) explains 10.60%; and factor 3 (Eigenvalue = 1.19) explains 9.17%. Past research has revealed both 2-factor (Maloney et al., 2012; Manapat et al., 2021) and 3-factor structures (De Ridder et al., 2011).

^8^Note that endogenous treatment regression analysis results do not render statistics that can be used for effect size calculation such as R-squares. Hence, no effect size is provided for the effects shown in the endogenous treatment regressions throughout the manuscript. However, to obtain an approximate effect size, we calculated effect sizes using equivalent OLS regressions and report them in the OLS regressions results in the Supplementary Material.

^9^Factor analysis was again conducted with the measured scales in the same manner as in Study 1. Again, the results are consistent with the known structures of the scales. For the 10-item dietary restraint scale, Barlett’s test of sphericity was significant (χ^2^(45) = 713.07, *p* < 0.001) and the two-factor structure was shown (goodness-of-fit χ^2^(26) = 40.45, *p* = 0.035): Factor 1 (Eigenvalue = 3.30) explains 32.99% of variance and factor 2 (Eigenvalue = 1.44) does 14.39%. This is consistent with prior literature as well as the results of Study 1. For the 11-item consumer impulsivity scale, Barlett’s test of sphericity was significant (χ^2^(55) = 1395.26, *p* < 0.001) and the two-factor structure was shown (goodness-of-fit χ^2^(34) = 130.66, *p* < 0.001): Factor 1 (Eigenvalue = 4.18) explains 37.97% of variance and factor 2 (Eigenvalue = 1.70) does 15.44%. Again, this is consistent with Study 1 and prior literature. Lastly, for the 13-item brief self-control scale, Barlett’s test of sphericity was significant (χ^2^(78) = 1117.95, *p* < 0.001) and the three-factor structure emerged (goodness-of-fit χ^2^(42) = 70.88, *p* = 0.004): Factor 1 (Eigenvalue = 4.19) explains 32.20% of variance, factor 2 (Eigenvalue = 1.46) does 11.22%; and factor 3 (Eigenvalue = 1.22) explains 9.35%. This is similar to the structure found in Study 1 and De Ridder et al. (2011).

^10^Detailed results of the analysis with brief self-control scale and consumer impulsivity scale are not reported here for they are no longer the focus of this study and there were no significant effects with these scales.
