# Supplementary material for: Dynamics of self-control during choice and post-choice consumption quantity
Source: Front Psychol. 2024 Jun 3;15:1238780. doi: 10.3389/fpsyg.2024.1238780 (PMC11181909; doi:10.3389/fpsyg.2024.1238780)
Supplement: Supplementary file 1 [file Data_Sheet_1.docx]

Supplementary Material

Dynamics of self-control during choice and post-choice consumption quantity

Ga-Eun (Grace) Oh*, Anirban Mukhopadhyay

*** Correspondence:** Corresponding Author: [geoh@connect.ust.hk](mailto:geoh@connect.ust.hk)

**Table of contents**

**1 Supplementary Figures and Tables**

**1.1. Supplementary Figures**

**Supplementary Figure 1.** Real Options Presented for Choice in Study 1.

**Supplementary Figure 2.** Calorie Intake as a Function of Cognitive Load, Food Choice, and Dietary Restraint in Study 1.

**Supplementary Figure 3.** Real Options Presented for Choice in Study 2.

**Supplementary Figure 4.** The Setting for the Lexical Decision Task in Study 2.

**1.2. Supplementary Tables**

**Supplementary Table 1**. Descriptive Statistics and Correlations of Variables in Study 1.

**Supplementary Table 2**. Endogenous Treatment Regression for Quantity Consumed in Study 1.

**Supplementary Table 3**. OLS Regression for Quantity Consumed in Study 1.

**Supplementary Table 4**. Endogenous Treatment Regression for Calorie Intake in Study 1.

**Supplementary Table 5**. OLS Regression for Calorie Intake in Study 1.

**Supplementary Table 6**. Descriptive Statistics and Correlations of Variables in Study 2.

**Supplementary Table 7**. Endogenous Treatment Regression for Facilitation Score for a Self-Control Goal in Study 2.

**Supplementary Table 8**. Endogenous Treatment Regression for Facilitation Score for an Indulgence Goal in Study 2.

**Supplementary Table 9**. OLS Regressions for Facilitation Scores for Self-Control and Indulgence Goals in Study 2.

**1 Supplementary Figures and Tables**

**1.1. Supplementary Figures**

**Supplementary Figure 1. Real Options Presented for Choice in Study 1**


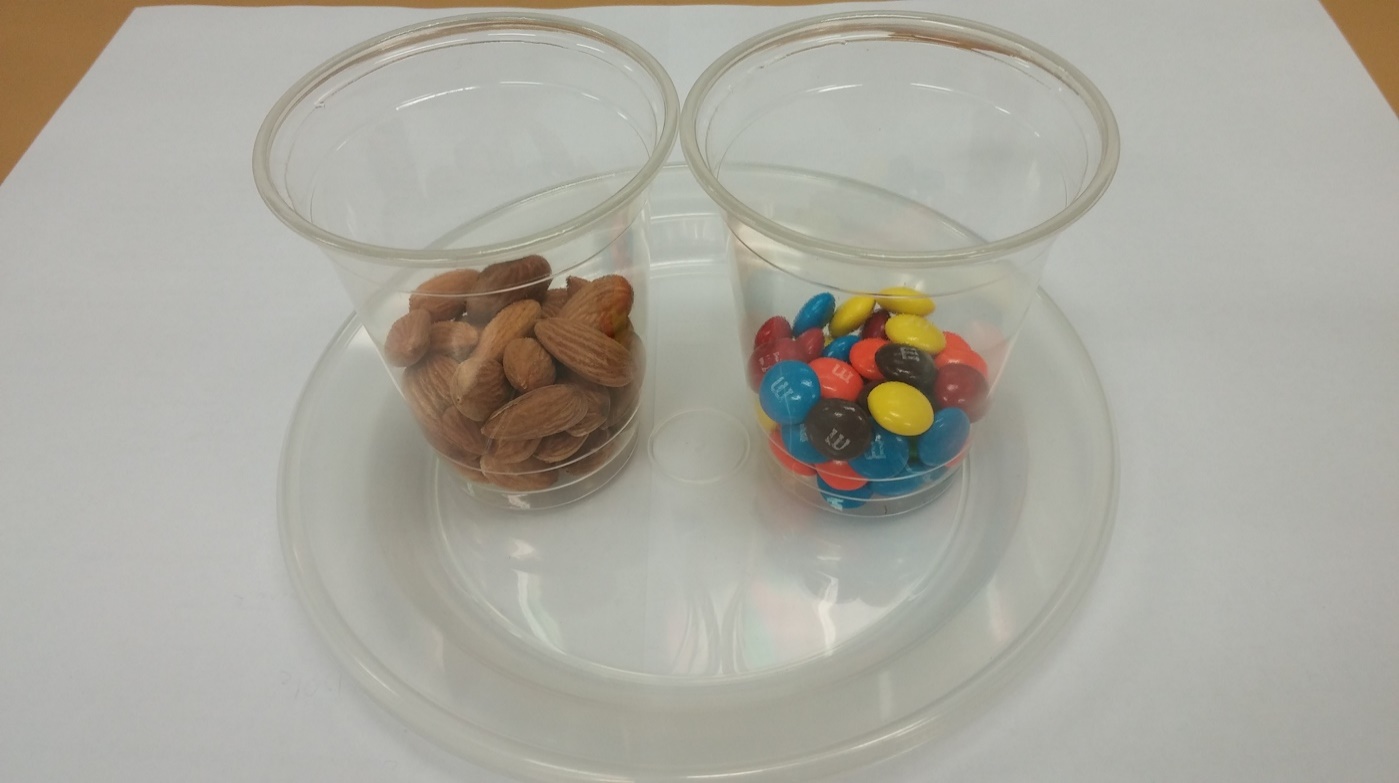


**Supplementary Figure 2. Calorie Intake as a Function of Cognitive Load, Food Choice, and Dietary Restraint in Study 1**

(A) High cognitive load


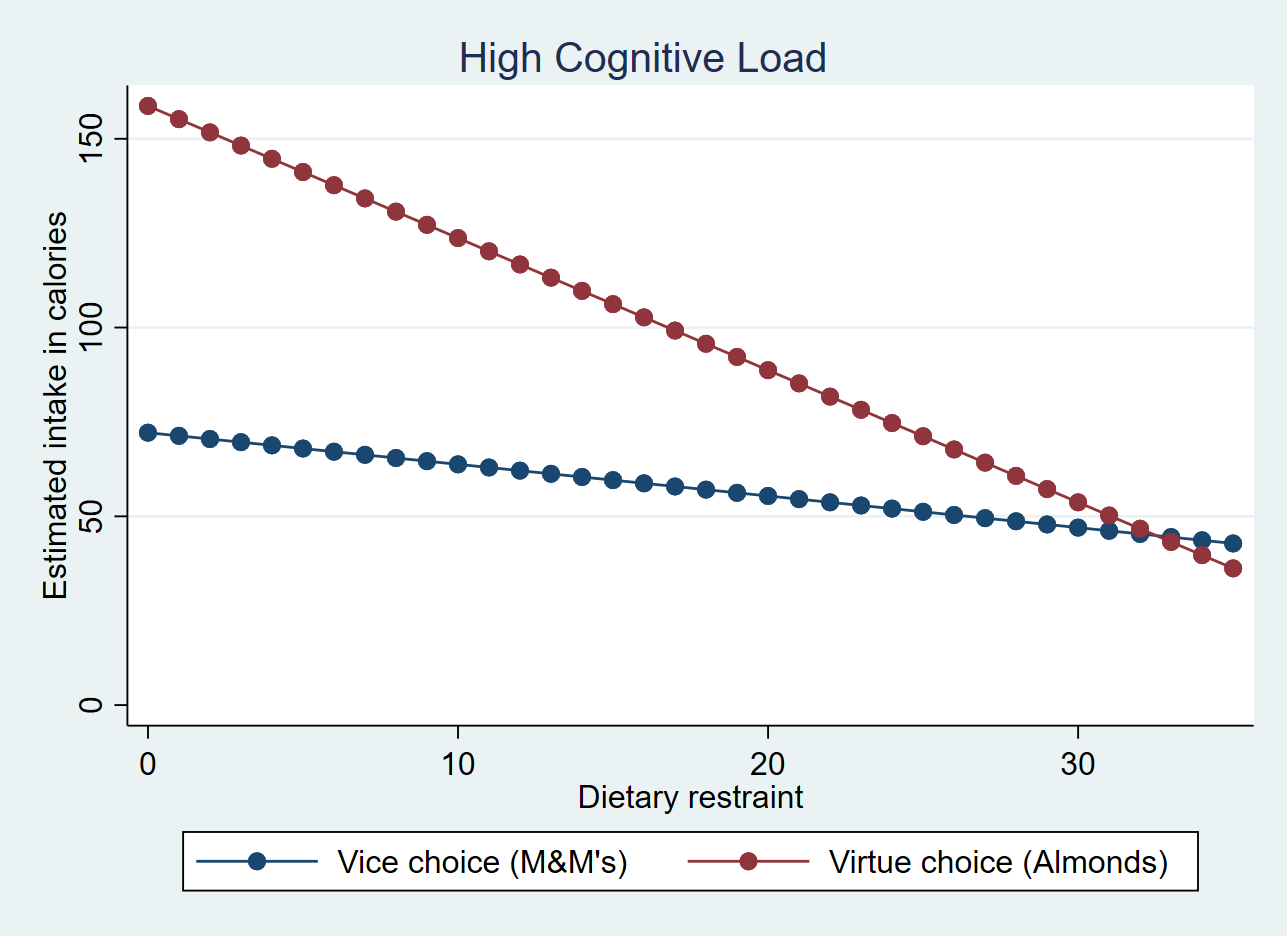


(B) Low cognitive load


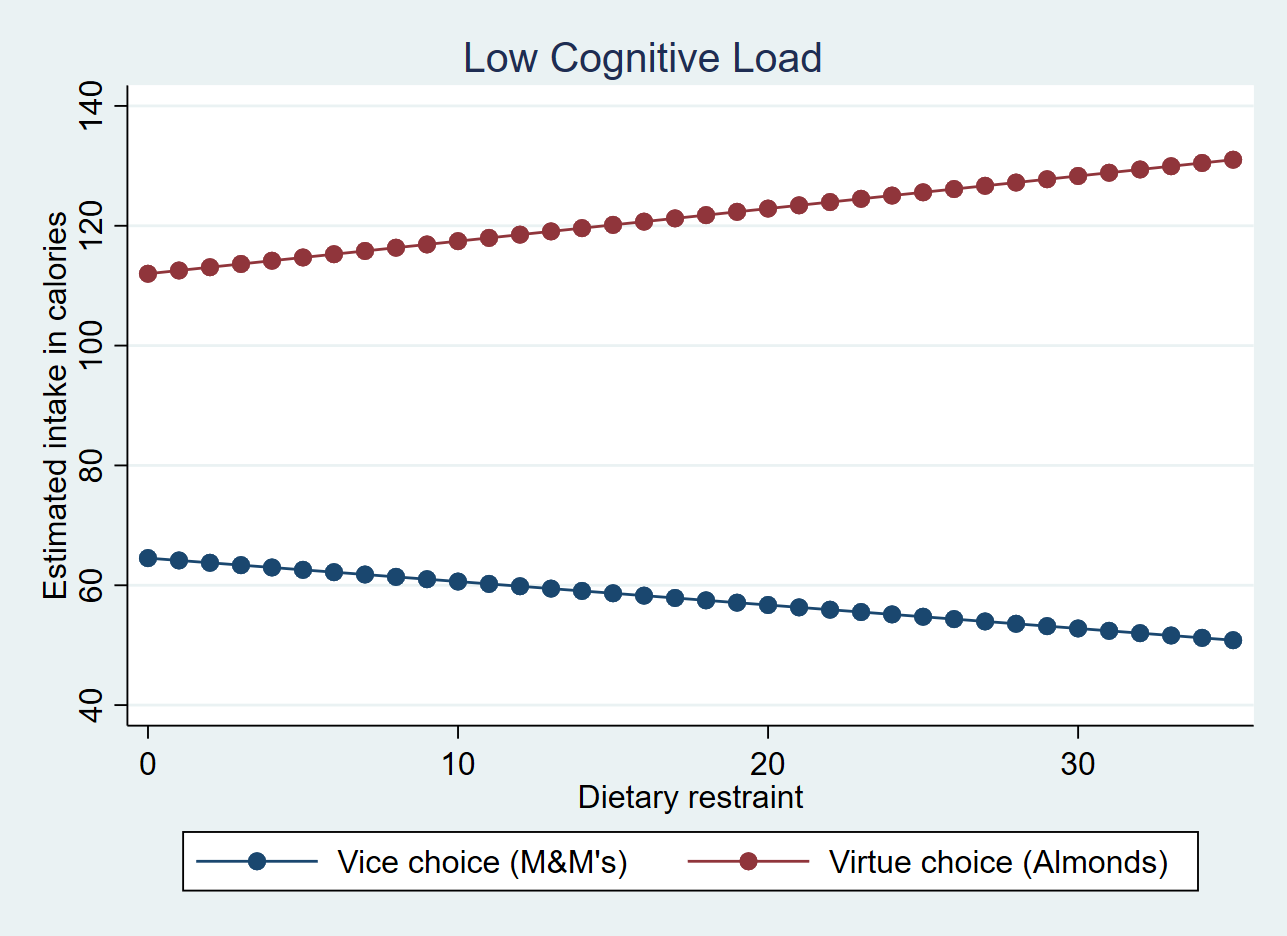


*Note*. Estimates plotted using raw dietary restraint scores (range: 0-35).

**Supplementary Figure 3. Real Options Presented for Choice in Study 2**


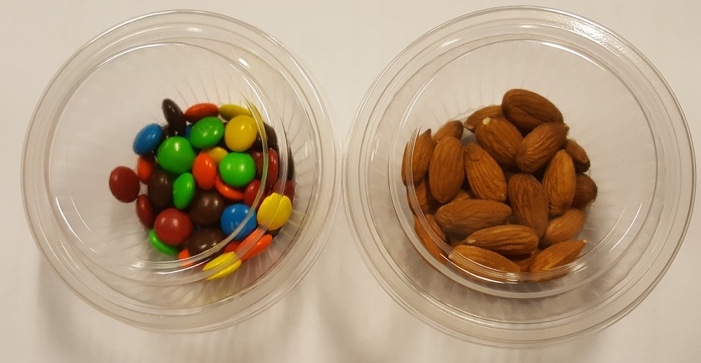


**Supplementary Figure 4. The Setting for the Lexical Decision Task in Study 2**


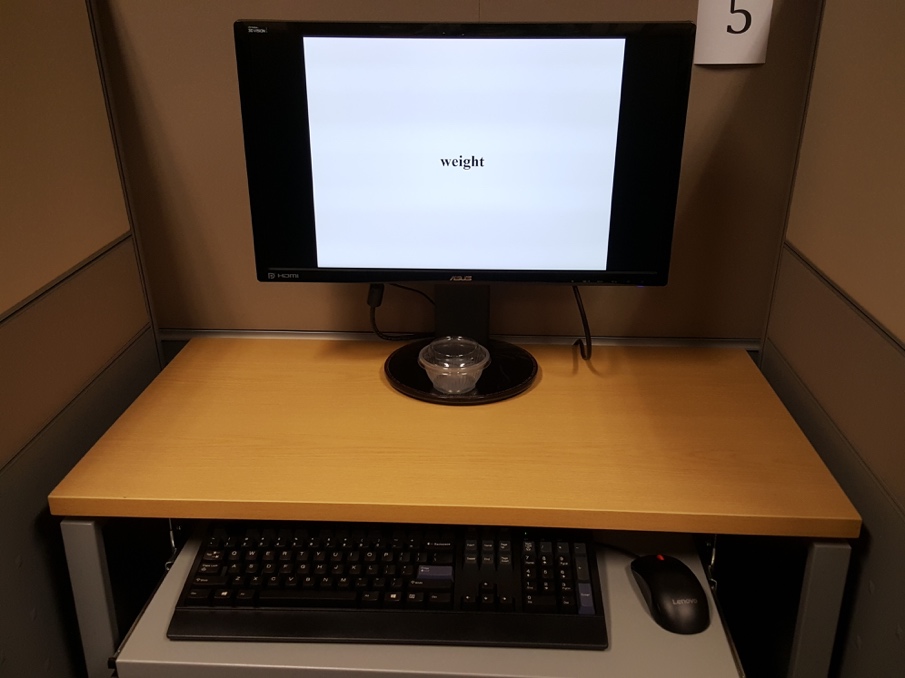


**1.2. Supplementary Tables**

**Supplementary Table 1. Descriptive Statistics and Correlations of Variables in Study 1**

|  | Mean | SD | 2. | 3. | 4. | 5. | 6. | 7. |
| --- | --- | --- | --- | --- | --- | --- | --- | --- |
| 1. Quantity consumed (in grams) | 15.60 | 9.78 | .994*** | –.05 | –.02 | –.06 | .31*** | .15*** |
| 2. Calorie intake | 79.72 | 50.12 | -- | –.06 | .07 | –.06 | .31*** | .13*** |
| 3. Cognitive load^a^ | .50 | .50 |  | -- | –.09* | –.03 | .01 | –.04 |
| 4. Food choice^b^ | 0.35 | 0.48 |  |  | -- | .10* | –.03 | –.13*** |
| 5. Dietary restraint | 12.77 | 5.47 |  |  |  | -- | –.06 | –.01 |
| 6. Subjective hunger | 3.79 | 1.78 |  |  |  |  | -- | .10* |
| 7. Tastiness of sampled food | 5.21 | 1.10 |  |  |  |  |  |  |

^a^coded as 0 = low cognitive load; 1 = high cognitive load.

^b^coded as 0 = a choice of a vice; 1 = a choice of a virtue.

* *p* < .05; ***p* < .01; ****p* < .001.**Supplementary Table 2. Endogenous Treatment Regression for Quantity Consumed in Study 1**

Linear Regression with Endogenous Treatment Model

Model Wald *χ*^2^(9) = 178.52, *p* < .001

Outcome: Quantity consumed (in grams)

|  | Coefficient | SE | t | p |
| --- | --- | --- | --- | --- |
| Constant | –1.02 | 2.04 | –.50 | .618 |
| Cognitive load | .85 | 1.01 | .84 | .400 |
| Food choice | 14.68 | 2.13 | 6.89 | <.001 |
| Dietary restraint | –.50 | .80 | –.63 | .531 |
| Cognitive load x Food choice | –1.26 | 1.54 | –.82 | .411 |
| Food choice x Dietary restraint | 1.13 | 1.09 | 1.04 | .299 |
| Cognitive load x Dietary restraint | –.79 | 1.08 | –.73 | .466 |
| Cognitive load x Food choice x Dietary restraint | –4.01 | 1.51 | –2.66 | .008 |
| Subjective hunger | 1.60 | .19 | 8.30 | <.001 |
| Tastiness of sampled food | 1.01 | .33 | 3.07 | .002 |

Outcome: Food choice

|  | Coefficient | SE | t | p |
| --- | --- | --- | --- | --- |
| Constant | –.25 | .07 | –3.71 | <.001 |
| Cognitive load | –.26 | .10 | –2.66 | .008 |
| Dietary restraint | .01 | .07 | .12 | .905 |
| Cognitive load x Dietary restraint | .25 | .10 | 2.44 | .015 |

Wald test of independence of equations (rho = 0): Wald *χ^2^*(1) = 44.82, *p* < .001

|  | Coefficient | SE |  |  |
| --- | --- | --- | --- | --- |
| *ρ* | –.76 | .06 |  |  |
| *σ* | 11.21 | .60 |  |  |
| λ | –8.48 | 1.12 |  |  |

*Notes.* Cognitive load coded as 0 = low load; 1 = high load. Food choice coded as 0 = vice; 1 = virtue. Dietary restraint scale standardized.

**Supplementary Table 3. OLS Regression for Quantity Consumed in Study 1**

Outcome: Quantity consumed (in grams)

|  | Coefficient | SE | t | p | f^2^ |
| --- | --- | --- | --- | --- | --- |
| Constant | 4.43 | 1.95 | 2.27 | .024 |  |
| Cognitive load | –.35 | .89 | –.40 | .693 | .003 |
| Food choice | .88 | 1.03 | .85 | .395 | .001 |
| Dietary restraint | –.41 | .66 | –.62 | .539 | .004 |
| Cognitive load x Food choice | –1.39 | 1.52 | –.91 | .363 | .003 |
| Food choice x Dietary restraint | .94 | 1.00 | .94 | .348 | .000 |
| Cognitive load x Dietary restraint | .23 | .91 | .248 | .804 | .003 |
| Cognitive load x Food choice x Dietary restraint | –3.20 | 1.51 | –2.12 | .035 | .006 |
| Subjective hunger | 1.65 | .20 | 8.22 | <.001 | .107 |
| Tastiness of sampled food | .98 | .33 | 2.97 | .003 | .012 |

*Notes.* Cognitive load coded as 0 = low load; 1 = high load. Food choice coded as 0 = vice; 1 = virtue. Dietary restraint scale standardized.

**Supplementary Table 4. Endogenous Treatment Regression Model for Calorie Intake in Study 1**

Linear Regression with Endogenous Treatment Model

Model Wald *χ*^2^(9) = 112.14, *p* < .001

Outcome: Calorie intake

|  | Coefficient | SE | t | p |
| --- | --- | --- | --- | --- |
| Constant | 1.67 | 14.73 | .11 | .910 |
| Cognitive load | 1.91 | 5.35 | .36 | .722 |
| Food choice | 59.40 | 27.62 | 2.15 | .032 |
| Dietary restraint | –2.14 | 3.75 | –.57 | .569 |
| Cognitive load x Food choice | –6.83 | 7.98 | –.86 | .392 |
| Food choice x Dietary restraint | 5.11 | 5.62 | .91 | .363 |
| Cognitive load x Dietary restraint | –2.44 | 5.53 | –.44 | .659 |
| Cognitive load x Food choice x Dietary restraint | –19.66 | 7.99 | –2.46 | .014 |
| Subjective hunger | 8.30 | 1.01 | 8.25 | <.001 |
| Tastiness of sampled food | 5.07 | 1.73 | 2.92 | .003 |

Outcome: Food choice

|  | Coefficient | SE | t | p |
| --- | --- | --- | --- | --- |
| Constant | –.26 | .07 | –3.75 | <.001 |
| Cognitive load | –.26 | .10 | –2.56 | .011 |
| Dietary restraint | .00 | .07 | .05 | .962 |
| Cognitive load x Dietary restraint | .27 | .10 | 2.60 | .009 |

Wald test of independence of equations (rho = 0): Wald *χ^2^*(1) = 2.44, *p* = .118

|  | Coefficient | SE |  |  |
| --- | --- | --- | --- | --- |
| *ρ* | –.54 | .27 |  |  |
| *σ* | 51.33 | 5.57 |  |  |
| λ | –27.79 | 17.01 |  |  |

*Notes.* Cognitive load coded as 0 = low load; 1 = high load. Food choice coded as 0 = vice; 1 = virtue. Dietary restraint scale standardized.

**Supplementary Table 5. OLS Regression for Calorie Intake in Study 1**

Outcome: Calorie Intake

|  | Coefficient | SE | t | p | f^2^ |
| --- | --- | --- | --- | --- | --- |
| Constant | 19.52 | 9.99 | 1.95 | .051 |  |
| Cognitive load | –1.74 | 4.55 | –.38 | .703 | .004 |
| Food choice | 14.43 | 5.28 | 2.73 | .006 | .004 |
| Dietary restraint | –1.98 | 3.37 | –.59 | .558 | .004 |
| Cognitive load x Food choice | –7.98 | 7.79 | –1.03 | .306 | .004 |
| Food choice x Dietary restraint | 4.84 | 5.11 | .95 | .344 | .001 |
| Cognitive load x Dietary restraint | 1.12 | 4.64 | .24 | .810 | .003 |
| Cognitive load x Food choice x Dietary restraint | –17.49 | 7.73 | –2.26 | .024 | .006 |
| Subjective hunger | 8.36 | 1.03 | 8.12 | <.001 | .104 |
| Tastiness of sampled food | 5.00 | 1.68 | 2.98 | .003 | .012 |

*Notes.* Cognitive load coded as 0 = low load; 1 = high load. Food choice coded as 0 = vice; 1 = virtue. Dietary restraint scale standardized.

**Supplementary Table 6. Descriptive Statistics and Correlations of Variables in Study 2**

|  | Mean | SD | 1. | 2. | 3. | 4. |
| --- | --- | --- | --- | --- | --- | --- |
| 1. Facilitation score for self-control goal | –1.24 | 69.73 | -- | .23*** | –.01 | .03 |
| 2. Facilitation score for indulgence goal | –34.60 | 85.24 |  | -- | .03 | .04 |
| 3. Food choice^a^ | .31 | .46 |  |  | -- | .07 |
| 4. Dietary restraint | 12.54 | 5.53 |  |  |  | -- |

^a^coded as 0 = a choice of a vice; 1 = a choice of a virtue.

****p* < .001.

**Supplementary Table 7. Endogenous Treatment Regression for Facilitation Score for Self-Control Goal in Study 2**

Linear Regression with Endogenous Treatment Model

Model Wald *χ*^2^(3) = 19.84, *p* < .001

Outcome: Facilitation score for Self-Control Goal

|  | Coefficient | SE | t | p |
| --- | --- | --- | --- | --- |
| ***DV: Facilitation Score for Self-Control Goal*** | | | | |
| Constant | 31.06 | 8.89 | 3.49 | <.001 |
| Food choice | –106.61 | 28.16 | –.3.79 | <.001 |
| Dietary restraint | .64 | 4.36 | .15 | .884 |
| Food choice x Dietary restraint | 16.85 | 6.99 | 2.41 | .016 |

Outcome: Food choice

|  | Coefficient | SE | t | p |
| --- | --- | --- | --- | --- |
| Constant | –.49 | .07 | –6.90 | <.001 |
| Dietary restraint | .08 | .07 | 1.19 | .233 |

Wald test of independence of equations (rho = 0): Wald *χ^2^*(1) = 11.41, *p* < .001

|  | Coefficient | SE |  |  |
| --- | --- | --- | --- | --- |
| *ρ* | .75 | .13 |  |  |
| *σ* | 84.30 | 10.91 |  |  |
| λ | 63.54 | 18.44 |  |  |

*Notes.* Food choice coded as 0 = vice; 1 = virtue. Dietary restraint scale standardized.

**Supplementary Table 8. Endogenous Treatment Regression for Facilitation Score for Indulgence Goal in Study 2**

Linear Regression with Endogenous Treatment Model

Model Wald *χ*^2^(3) = 5.05, *p* < .168

Outcome: Facilitation score for Indulgence Goal

|  | Coefficient | SE | t | p |
| --- | --- | --- | --- | --- |
| ***DV: Facilitation Score for Indulgence Goal*** | | | | |
| Constant | –43.68 | 9.59 | –4.56 | <.001 |
| Food choice | 27.36 | 28.43 | 1.00 | .319 |
| Dietary restraint | –.53 | 5.31 | –.10 | .920 |
| Food choice x Dietary restraint | 10.39 | 7.98 | 1.30 | .19 |

Outcome: Food choice

|  | Coefficient | SE | t | p |
| --- | --- | --- | --- | --- |
| Constant | –.50 | .07 | –7.16 | <.001 |
| Dietary restraint | .09 | .07 | 1.35 | .178 |

Wald test of independence of equations (rho = 0): Wald *χ^2^*(1) = .91, *p* = .341

|  | Coefficient | SE |  |  |
| --- | --- | --- | --- | --- |
| *ρ* | –.18 | .18 |  |  |
| *σ* | 85.65 | 4.63 |  |  |
| λ | –15.00 | 15.59 |  |  |

*Notes.* Food choice coded as 0 = vice; 1 = virtue. Dietary restraint scale standardized.

**Supplementary Table 9. OLS Regressions for Facilitation Scores for Self-Control and Indulgence Goals in Study 2**

Outcome: Facilitation score

|  | Coefficient | SE | t | p | f^2^ |
| --- | --- | --- | --- | --- | --- |
| ***DV: Facilitation Score for Self-Control Goal*** | | | | |  |
| Constant | –1.05 | 4.46 | -.24 | .814 |  |
| Food choice | –2.18 | 8.06 | –.27 | .787 | .000 |
| Dietary restraint | –2.27 | 4.42 | –.51 | .609 | .001 |
| Food choice x Dietary restraint | 14.90 | 8.18 | 1.82 | .069 | .009 |
|  |  |  |  |  |  |
| ***DV: Facilitation Score for Indulgence Goal*** | | | | |  |
| Constant | –36.04 | 5.47 | –6.59 | <.001 |  |
| Food choice | 3.57 | 9.88 | .36 | .718 | .001 |
| Dietary restraint | .19 | 5.42 | .03 | .973 | .002 |
| Food choice x Dietary restraint | 10.71 | 10.03 | 1.07 | .286 | .003 |

*Notes.* Food choice coded as 0 = vice; 1 = virtue. Dietary restraint scale standardized.
